# Quality-space theory in olfaction

**DOI:** 10.3389/fpsyg.2014.00001

**Published:** 2014-01-16

**Authors:** Benjamin D. Young, Andreas Keller, David Rosenthal

**Affiliations:** ^1^The Department of Cognitive and Brain Science, Ben-Gurion University of the NegevBeer-Sheva, Israel; ^2^Philosophy Program, Graduate Center, City University of New YorkNew York, NY, USA; ^3^Philosophy Program and Concentration in Cognitive Science, Graduate Center, City University of New YorkNew York, NY, USA

**Keywords:** quality-space theory, quality-space, olfaction, mental qualities, phenomenology, perceptual qualities, consciousness

## Abstract

Quality-space theory (QST) explains the nature of the mental qualities distinctive of perceptual states by appeal to their role in perceiving. QST is typically described in terms of the mental qualities that pertain to color. Here we apply QST to the olfactory modalities. Olfaction is in various respects more complex than vision, and so provides a useful test case for QST. To determine whether QST can deal with the challenges olfaction presents, we show how a quality space (QS) could be constructed relying on olfactory perceptible properties and the olfactory mental qualities then defined by appeal to that QS of olfactory perceptible properties. We also consider how to delimit the olfactory QS from other modalities. We further apply QST to the role that experience plays in refining our olfactory discriminative abilities and the occurrence of olfactory mental qualities in non-conscious olfactory states. QST is shown to be fully applicable to and useful for understanding the complex domain of olfaction.

## QUALITY-SPACE THEORY

Conscious perceiving always subjectively involves conscious qualitative character. Conscious vision involves qualities of color and of visible shape, size, and location; conscious audition involves qualities of pitch, loudness, timbre, and audible location; and conscious olfaction involves mental qualities that correspond to the various odorants and, possibly, qualities that correspond to their locations.

Perceiving occurs not only consciously, but without being conscious as well, in masked priming and other forms of subliminal perceiving (e.g., Marcel, 1983; Sobel et al., 1999; Breitmeyer and Öğmen, 2006; Öğmen and Breitmeyer, 2006; Zucco et al., 2013). In the conscious cases, we know about the qualitative character of perceptual states subjectively, by how they appear to consciousness. So many are tempted to conclude that when perceptual states do occur without being conscious, there is no way to know about their qualitative character, and hence no qualitative character in that case to know about.

This line of thought has led many in philosophy to deny that non-conscious perceptual states exhibit any qualitative character, properly so called (e.g., Nagel, 1974). It has also led many in philosophy to see the qualitative character of perceptual states as deeply problematic. It has been argued that neural processes cannot constitute or give rise to conscious qualitative character (Chalmers, 1996), or that, if they can, we cannot in any case explain how that can be (Levine, 2001). And it has been urged that since we know about qualitative character only by way of consciousness, i.e., only by first-person access, the specific types of mental quality that figure in perceiving a particular physical property might differ from one individual to another in ways that are empirically undetectable (e.g., Shoemaker, 1975/1984). Carrying this to an extreme, it has been held that an individual that is physically indistinguishable from us and functions in ways perceptually indistinguishable from us might nonetheless lack conscious mental qualities (Chalmers, 1996). And many not in philosophy have found these conclusions tempting as well.

Concern with these apparent conundra has generated a large literature in philosophy, with many arguing that these problems cannot be sidestepped or resolved and others proposing solutions that seldom gain lasting wide adherence. But both sides in this debate typically operate on the assumption that since qualitative character occurs only in conscious perceiving, that we can know about qualitative character only by way of subjective, first-person access.

The demonstrable occurrence of perceiving that is not conscious forces reexamination of the assumption that subliminal and other non-conscious perceiving is actually devoid of qualitative character, and that non-conscious perceiving discharges its psychological and biological function without benefit of mental qualities. And any such reexamination shows that it is by no means obvious that non-conscious perceiving lacks qualitative character.

For one thing, we describe non-conscious perceptual states in the same qualitative terms we use to taxonomize conscious perceptual states. That is evident, for example, in experimental work on masked priming. Participants visually but non-consciously perceive stimuli in respect of their colors and shapes; we classify the non-conscious visual states in respect of those qualitative terms despite those states’ not being conscious. More important, it is clear in conscious perceiving that differences in qualitative character are responsible for the discriminative ability characteristic of perceiving; in conscious perceiving it is plain that we would be unable to distinguish color, shapes, sounds, and odors without our conscious perceptual states’ differing in qualitative character in ways that make such discriminations possible. But unconscious perceiving also enables the discrimination of various environmental properties, indeed largely the same discriminations we make in conscious perceiving. In addition, when experimental participants guess about degraded stimuli (Cheesman and Merikle, 1986; Merikle, 1992; Dienes and Seth, 2010), where guessing taken to be an indication that the perceiving is not conscious, they guess about colors and other qualitative character, indicating non-conscious qualitative states that reflect those qualitative properties. So it is natural to infer that differences in qualitative character in unconscious perceiving as well, enabling us to make those discriminations.

Consider the visual case. Consciously seeing something red is being in a visual state that is more like seeing orange than like seeing blue or green; similarly, subliminally seeing something red is being in just such a state, except the state is not conscious. Without some compelling independent reason, reserving the notion of mental qualitative character for conscious states is an arbitrary and unwarranted stipulation from a time when the occurrence of non-conscious perceiving was not recognized. And as with empirical and theoretical issues generally, we must rest with the strongest considerations available to us.

The assumption that non-conscious perceiving lacks qualitative character seems tempting only if one sees no way to learn about and describe mental qualities except by how they subjectively appear in consciousness. But it is worth stressing that appeal to that assumption begs the question at hand. If unconscious perceiving lacks qualitative character, then we will have access to qualitative character primarily, and perhaps exclusively, by way of consciousness. Some independent consideration is needed to settle the issue about whether qualitative character ever occurs without being conscious.

Quality-space theory (QST; Rosenthal, 1991, 1999, 2005, 2010, in press; Clark, 1993) offers just such an independent reason. It constructs an alternative to the exclusive reliance on subjective consciousness, by explaining the nature of mental qualities by appeal to their role in perceiving. Since perceiving can occur without being conscious, QST provides an explanation of qualitative character that applies to perceiving independent of whether it is conscious, and hence without in any way relying on conscious access to qualitative character.

The core idea of QST rests on the discriminative function of perception mentioned above. Perceiving always involves discrimination of properties accessible by a particular sensory modality. And to discriminate two properties, p_1_ and p_2_, a creature must be able to be in psychological state of two distinct types, each type corresponding in some suitably differential way to one of the two perceptible properties. The two types of perceptual state must differ in respect of some psychologically relevant properties.

The conscious perceptual states that enable discrimination of perceptible properties differ in respect of qualitative character. So it is natural to identify as mental qualities the differential psychological properties that enable discrimination of perceptible properties, whether that discrimination occurs consciously or not. Perceptual states enable discrimination of perceptible properties by differing in respect of mental quality. Mental qualities are the psychological properties in virtue of which a creature can distinguish among the various properties accessible to each perceptual modality.

One can measure discriminative ability by testing for just-noticeable differences (JNDs) between barely discriminable properties for a particular modality. Methodological issues arise because discriminability is not transitive; p_1_ may be just noticeably different from p_2_and p_2_ from p_3_ even though p_1_ and p_3_ are indistinguishable (e.g., Goodman, 1951). But despite that, one can use JNDs to construct a quality space (QS) that represents all the discriminations that a particular individual can make among the perceptible properties accessible by a particular modality. The dimensions of this space will emerge as needed; it may be that though p_1_, p_2_, and p_3_ are just noticeably different in a linear fashion, several other perceptible properties are just noticeably different from each of p_1_, p_2_, and p_3_, in ways that induce a new dimension for the QS of the perceptible properties accessible by that modality (Clark, 1993, pp. 84–89).

The QSs constructed in this way describe discriminability of the perceptible properties accessible by a particular modality. But because mental qualities are the differential psychological properties of states that enable such discriminations, the very same space will also capture the differences and similarities among those mental qualities. So that QS describes and explains what mental qualities are, and how we taxonomize them by type. And since the relevant discriminability among stimuli accessible by a particular modality occur in both conscious and non-conscious perceiving, a QST account of mental qualities is independent of how they present themselves subjectively to consciousness.

Indeed, one can establish non-conscious JNDs. As noted earlier, when stimuli are degraded, subjects’ JND judgments remain accurate even when subjects take themselves to be merely guessing (Cheesman and Merikle, 1986; Merikle, 1992; Dienes and Seth, 2010). Since taking oneself to guess indicates that one is not consciously aware of JNDs, it reflects perceptual states that are not conscious.

The QS of perceptible properties matches that of the mental qualities that enable discrimination among those perceptible properties. One might conclude that such a match cannot be established without subjective awareness of the relevant mental qualities, and so QST cannot after all apply to non-conscious perceiving. This is a mistake. The match between QSs is established not by comparing the space of discriminable perceptible properties with the space of corresponding mental qualities. Rather, it is established by extrapolating from the space of perceptible properties to that of mental qualities. That extrapolation is an inference to the best explanation of what makes possible the discriminations used to construct the QS of perceptible properties.

Constructing QSs using discriminative abilities does not appeal to normal or typical conditions of perceiving. JNDs are used to construct the QS of perceptible properties; so optimal conditions for each individual tested are what matter. Moreover, the QS of perceptible properties will not in general reflect the physical properties of the stimuli, since the space is constructed not by appeal to the physical nature of the stimuli, but to how an individual discriminates among them.

A dramatic case of this occurs with color, where there are different wavelength distributions that are perceptually indistinguishable. So on QST, these different wavelength distributions result in the same mental qualities. If the space that determined mental qualities were constructed from the physical properties of stimuli, such stimuli would determine distinct mental qualities. On a space of perceptible properties constructed from discriminability, as determined by JNDs, these stimuli occupy identical positions, and so determine identical mental qualities. But it is important that this kind of phenomenon apart, perceptible properties are grouped for purposes of QST not by appeal to their physical characteristics, but by the ability of perceivers to discriminate among them.

Perceptual acuity can differ not only between individuals but also within an individual over time. Perceptual acuity can improve, e.g., by perceptual learning or maturation, which results in an enhanced or more fine-grained space of discriminable stimuli, and a correspondingly enlarged or more fine-grained space of mental qualities. And though one tests individuals, averaging over members of a species will capture average discriminability for that species. There are other refinements (Rosenthal, 2005, ch. 7, 2010, in press), but these will not bear on our purposes here.

Each stimulus type an individual can discriminate from its neighbors is physically distinct from other stimuli; so it is describable on its own, independent of any others. But because the QS of perceptible properties is constructed from the discriminability relations that JNDs deliver, the locations in the space of all the perceptible properties is determined relative to the location of other perceptible properties.

No perceptible property has a fixed position independent of its discriminability relations to the others. The space is not constructed by first having some fixed locations for some privileged perceptible properties and locating others relative to them; it is constructed by determining the relations of discriminability among all the properties accessible by a particular modality. So the theory represents each type of perceptible property comparatively, by appeal to which other properties it is discriminable from. And QST accordingly also represents each type of mental quality comparatively, by appeal to relative discriminability of stimuli.

By contrast, theories that explain the nature of mental qualities by appeal to the way they subjectively appear in consciousness result in a non-comparative, non-relational taxonomy of the types of mental quality, typing each independently of all others. Relying on first-person, subjective access to mental qualities tends to result in a non-comparative taxonomy, since consciousness by itself can access only the token mental qualities, independent of how they are typed. Having picked out token mental qualities, subjective awareness can then compare them; but subjective awareness must on such an account be able to individuate token mental qualities independent of any such comparisons.

It is this feature of the reliance on consciousness in explaining what mental qualities are that makes it appealing to imagine that one person’s mental quality on seeing a red stimulus could undetectably be the same as another person’s on seeing a green stimulus. If first-person access could trump everything else we know about mental qualities, there would be no way to exclude that strange apparent possibility.

QST, by contrast, precludes such undetectable inversion. The QS for every known perceptual modality is asymmetric (see, e.g., Kuehni, 1998, 2003, ch. 6, 2005, ch. 6; Ramanath et al., 2004). Even the one-dimensional space of grayscale shades is asymmetric, due to the anchoring effect in which the lightest shade in any local framework appears to be pure while (Gilchrist, 2006). And though sufficiently detailed work on the discriminability space of other perceptible properties has not been done, there is no reason to expect that the resulting QSs will turn out in any such case to be symmetrical.

The mental qualities that pertain to visible colors are a useful initial test case for QST, partly because the dimensions of the QS are few in number and well understood, and partly because color plays such a prominent role in our conscious experience. Individuals discriminate among colors along various dimensions, which turn out to correspond to the standard properties of hue, saturation, and brightness. When individuals are tested for discrimination of neighboring color stimuli, it turns out that these are dimensions that emerge, resulting in a three-dimensional QS of discriminable color properties (e.g., CIE, 1932; Clark, 1993).

QST builds from a QS of perceptible colors, and extrapolates to determine the various types of mental color quality in terms of their relative location in a QS isomorphic to that of the perceptible colors. But it may be tempting to suppose that there are after all perceptual primitives for color, fixed not comparatively as in QST, but in an absolute, non-comparative way.

Focusing on how we consciously perceive things makes it inviting to posit perceptual primitives that operate in a non-comparative way. Subjective awareness of mental color qualities makes it seem that each of the colors is what it is just on its own, and not as a function of relations with others. Consciousness can compare the various mental color qualities, but only by treating each as independently fixed. So subjective consciousness cannot represent the mental qualities as fixed in a comparative, relational way.

But since perceiving also occurs without being conscious, if there were perceptual primitives, they would be common to conscious and non-conscious perceiving. So the way consciousness represents mental qualities cannot by itself ground the positing of perceptual primitives. The JNDs used to construct QSs, by contrast, fix perceptible properties by relative discriminability and hence comparatively; the corresponding mental qualities follow suit.

One might argue that a comparative taxonomy of mental qualities cannot be correct if subjective awareness does not present them comparatively. But it is far from obvious that subjective awareness does present mental qualities non-comparatively. We have subjective access to each token mental color quality, for example, in respect of its comparative location in our field of vision, as fixed by the boundaries of that field, beyond which there are no more mental color qualities.

Consciousness aside, however, perhaps the relevant neural machinery fixes some perceptual primitives in an absolute, non-comparative way. In color vision, appeal to opponent-processing theory (Hurvich and Jameson, 1957) may seem to underwrite such perceptual primitives. On opponent-processing theory, channels in the optic nerve code retinal color information as relative strengths of opponent colors, red vs. green, blue vs. yellow, and black vs. white.

But even if the opponent-processing hypothesis is correct, it would not undermine QST, which seeks to explain not the mechanisms of perceiving, but the qualitative character of perceptual states that such perceptual machinery subserves. And the mental qualities distinctive of each modality are a matter of the discriminative ability that the perceptual apparatus, whatever it may be, enables; they are properties of perceptual states in virtue of which an individual can discriminate instances of a range of perceptible properties. Even if particular types of stimulus are especially salient for the perceptual apparatus of a particular modality, such perceptual primitives do not figure in fixing the types of mental quality for that modality.

There is a striking illustration of how mental qualities may depart from what underlying perceptual mechanisms seem to dictate. On opponent-processing theory, seeing red and seeing green each result from outweighing the opponent color in the red–green channel; similarly for blue and yellow. So the theory should preclude so-called forbidden colors, such as reddish green and as yellowish blue.

But image stabilization, in which a retinal image is made to hold constant despite saccading, can produce cortical filling in that leads subjects presented with adjacent red and green stripes to report seeing reddish green; similarly for yellow and blue (Crane and Piantanida, 1983; Billock et al., 2001). These findings do not undermine opponent-processing theory, since opponent processing would occur in optic-nerve channels prior to the cortical filling in that results in these subjective experiences. But they do show that individuation of mental-quality types is not settled by appeal to underlying perceptual mechanisms. The typing of mental qualities rests on discriminability of one perceptible property from another.

Colors are not the only perceptible properties accessible by vision; vision also represents spatial properties of size, shape, and location. It captures these spatial properties as boundaries of discriminable colors; without discriminable differences in color, including the achromatic colors (black, gray, white), vision could not access size, shape, or location. And as just noted, some retinal motion relative to visual stimuli is needed for the normal visual perception of spatial boundaries, i.e., visible sizes, shapes, and locations QST handles the spatial mental qualities as it does other mental qualities. One can test for JNDs of visible shapes, sizes, and locations, and collate the results in QSs of those visible properties. And since mental qualities are the properties of perceptual states that enable perceptual discrimination, the QSs of visible shapes, sizes, and locations also determine the mental visual qualities of shapes, sizes, and locations (Meehan, 2001; Rosenthal, 2001, in press).

Perceptible objects have sizes, shapes, and locations independent of being perceived, and hence independent of the modality by which those spatial properties are perceived. But the mental qualities that pertain to these spatial perceptible properties are tied to particular modalities. Vision accesses the physical location of things, e.g., by boundaries of discriminable colors, tactition by discriminable resistance, pressure (Kappers and Bergmann Tiest, 2013), and texture, and audition by stereo effects of discriminable sounds. So distinct testing of JNDs is needed to establish QSs that capture the spatial perceptible properties discriminable by each modality, and those distinct QSs will in turn then fix the spatial mental qualities for each modality.

QST fixes the mental qualities of both conscious and non-conscious perception. So the theory cannot by itself explain how conscious perceiving differs from perceiving that is not conscious; an additional theory is needed to do that. If an individual is in a mental state of some type but is wholly unaware of being in that state, that shows that the state is not conscious; this test dominates work in experimental psychology and as well as our common sense views about conscious states. So a state is conscious only if the individual is aware of it in some suitable way. A successful explanation of what is distinctive of conscious perceiving differs will doubtless proceed along such lines (Rosenthal, 2004, 2005).

Since QST is not a theory of the difference between conscious and non-conscious states, the main theories of consciousness are not in direct competition with QST. Indeed, there are few if any theories that compete with QST in directly addressing the nature of mental qualities, as against mental representation more generally. The main alternative views are those that hold that our knowledge about qualitative character is limited to what subjective awareness reveals (e.g., Nagel, 1974; Kripke, 1980; Block, 1995; Chalmers, 1996; Levine, 2001). For further extended arguments against these views and their variants (see Rosenthal, 2010), which also advances compelling general considerations to think that QST is correct.

In this paper we do not attempt a comprehensive review of QST and the competing theories. Instead, we want to test if QST can deal with the challenges that olfaction presents. Each modality may raise its own issues about the dimensions of the relevant QSs, the possibility of perceptual primitives, the contrast between QST and an exclusively first-person, consciousness-based approach to mental qualities, the nature of mental qualities if any that occur in connection with spatial perception, and others. Here we use olfaction as an especially challenging test case for the theory.

## THE OLFACTORY QUALITY SPACE

The color QS based on JNDs is well-established. However, in other modalities much less progress has been made. We are, for example, not aware of any attempt to construct an olfactory QS based on JNDs. Furthermore, so far all attempts to arrange olfactory qualities based on other aspects of perception have failed (Berglund and Höglund, 2012; Kaeppler and Mueller, 2013), casting some doubt on whether there is any olfactory QS analogous to the color QS. However, there are good reasons to believe that there is such an olfactory QS and that the reason why it has not been described yet is that it is much more complex than the three-dimensional color QS.

Most attempts to establish an odor QS have been attempts to arrange individual odorous molecules (benzaldehyde, hexanal, vanillin, and so on) in a perceptual space based on the similarities in their perceived smell (Wise et al., 2000). Such a space would only cover a very small fraction of all olfactory qualities because mixtures of odorous molecules frequently have qualities that are different from the qualities of its components. No two mixtures of different odorous molecules that are perceptually indistinguishable have so far been identified. Furthermore, the use of a small number of odorous stimuli may reflect the idea that there are some perceptual primitives, which runs counter to the methodology of QST.

Because of the unique perceptual properties of each mixture, there is no easy way to use the space of the perceived qualities of individual odorous molecules as a basis to construct an olfactory QS that includes all olfactory qualities, including those found only in mixtures. The space of the perceived olfactory qualities of molecules would have to have the feature that no line connecting the perceptual qualities of two molecules crosses another such line (as this would be a case of two mixtures that are perceptually indistinguishable).

To construct an olfactory QS that covers all olfactory perceptual qualities, one has therefore to include the olfactory qualities of mixtures, which are also the qualities we are familiar with because the smells encountered in nature are almost always mixtures. The characteristic scent of a rose, for example, is produced by a mixture of 275 different odorous molecules (Ohloff, 1994, pp. 154–158). An olfactory QS of odor mixtures can be constructed based on JNDs by gradually altering the ratios of the components of one mixture until it becomes an olfactory stimulus distinguishable from the original.

It is easy to see that the QS constructed in this way would have to accommodate a very large number of distinguishable olfactory sensory qualities. There are 166 billion molecules with 17 or less atoms (Ruddigkeit et al., 2012) and a large majority of those that have been studied have an odor. Almost all these odorous molecules have a smell that can be distinguished from the smell of all other odorous molecules (Laska and Teubner, 1999a,b). The only instances of two different odorous molecules that have indistinguishable smells are certain pairs of enantiomers (mirror-symmetric molecules; Laska and Teubner, 1999b; Laska, 2004) and some pairs of molecules that differ only in that the hydrogen atoms have been replaced by deuterium atoms (Keller and Vosshall, 2004). Other pairs of enantiomers and pairs of molecules that differ only in hydrogen isotopes can be distinguished (Laska and Teubner, 1999b; Laska, 2004; Gane et al., 2013).

Odorous molecules can also be mixed in different combinations and ratios, further increasing the large number of possible olfactory stimuli. Only a very small fraction of these mixtures has ever been studied, but the fact that among those mixtures that have been studied there are none that are indistinguishable from others shows that many mixtures have unique olfactory qualities, and so would occupy a distinct locution in an olfactory QS.

On the other hand, there is independent reason to believe that not all mixtures have a unique smell. In one of the most significant recent discoveries in olfactory psychophysics, Tali Weiss and colleagues showed that mixtures with many components converge perceptually. This means that mixtures of random odorous molecules with a large enough number of components smell similar and share an olfactory quality that has been called “olfactory white” (Weiss et al., 2012). The reason why the complex mixtures of odorous molecules that we encounter when we smell roses or coffee do not smell similar is because the components of these mixtures are not a random sampling of odorous molecules and because in these naturally occurring mixtures there are some components represented at much higher intensity than the majority of the components. How many components are necessary in mixtures to render them indistinguishable from one another is not yet known, but on average, mixtures of 30 or 60 components can still be discriminated (Weiss et al., 2012).

These considerations suggest that there are very many perceptible properties in the olfactory modality that can be distinguished by human subjects, and consequently very many olfactory mental qualities that we must posit as responsible for such fine-grained olfactory discriminability ability. One thousand different odorous molecules can be mixed into 3e^+^^23^ different mixtures of 10 components. Even if each of those mixtures had the same smell as, on average, one trillion other mixtures, there would be 3e^+^^11^ different olfactory qualities. In comparison, there are approximately 340,000 tones (Stevens and Davis, 1938) and between 2.3 and 7.5 million colors (Nickerson and Newhall, 1943; Pointer and Attridge, 1998) that humans can distinguish.

From a biological perspective it would not be surprising if there were many more distinguishable odors than distinguishable colors. Color perception is mediated by differential activation of three different types of receptor whereas olfactory perception is mediated by differential activation of around 400 different types of receptors (Olender et al., 2012) and the possible combinations of 400 far exceed the possible combinations of three.

However, it is an interesting question how such a large number of perceptual qualities might be arranged into a QS. The two mathematical solutions to this problem, which are not mutually exclusive, are that the resolution along the dimensions of the space is very high or that there is a large number of dimensions in the olfactory QS. QSs are mathematical constructs that have whatever number of dimensions is needed to capture the discriminability relations of the relevant stimuli and the corresponding mental qualities. The QS for thermosensation for example seems to be one-dimensionality, along a dimension from cold to hot. Interestingly, this is so despite the fact that several types of receptors contribute to temperature sensation (Dhaka et al., 2006). The fact that the activity of several receptor types can result in a one-dimensional QS illustrates dramatically that QSs are not based the sensitivity of receptor types, but on JNDs. The color QS, like physical space, has three dimensions (hue, saturation, and brightness; Hilbert and Kalderon, 2000). That there are also three types of color receptors is a coincidence and irrelevant for the construction of the color QS. It has been suggested that the olfactory QS has a much higher dimensionality than QSs in other modalities (Berglund and Höglund, 2012; Auffarth, 2013).

It simply is not possible to arrange all olfactory qualities in a low-dimensional space. Qualities of odor mixtures (at least in most cases) are intermediary between the odor qualities of their components; the perceptual qualities of mixtures occupy the space delimited by their components in the QS (Wise and Cain, 2000; Berglund and Höglund, 2012). Two odorous molecules and all their mixtures fill a one-dimensional QS. Four molecules and their mixtures fill a two-dimensional QS, eight molecules and their mixtures a three-dimensional QS with the eight components of the mixture in the corners of a cube. To accommodate 1,000 odorous molecules and all their mixtures, approximately 10 dimensions would be required. To accommodate 100,000 odorous molecules and their mixtures, one would need a 17-dimensional space.

These considerations show that despite the failure of all previous attempts to do so, there is no reason to suppose that there is not an olfactory QS that is based on JNDs and methodologically on a par with the color QS. The difference between the two QSs is that the olfactory QS is larger and more complex than the color QS. The color QS arranges a few million qualities in a three-dimensional space whereas the olfactory QS arranges vastly more qualities in a substantially higher-dimensional space. The numerous failures to describe an olfactory QS are merely due to the extremely large dataset required to do so.

## THE OLFACTORY MODALITY

The odor QS represents olfactory qualities and the color QS represents color qualities, but how do we know which mental qualities are part of the color QS and which are part of the odor QS? Traditionally, sense modalities have been individuated by criteria, but there has been dispute over which criteria to use. The four main approaches current in the philosophical literature are to use a representational criterion, the phenomenal character criterion, the proximal stimulus criterion, or the sense-organ criterion (Grice, 1962; Macpherson, 2011). We will here discuss an alternative way of individuating modalities that does not depend on criteria, but instead individuates modalities based on the results of forced-choice discrimination tasks, the same methodology which is used to construct QSs.

The traditional criterion-based approaches largely agree in how they individuate vision and audition. However, with other modalities they often produce contradictory results. There is, for example, a type of molecular receptor (called TRPV1) that is sensitive both to hot temperature and to capsaicin, the pungent chemical found in chili peppers (Caterina et al., 1997). If the sensory-organ criterion is applied, capsaicin and heat are two stimuli in the same modality. If the stimulus criterion is applied, then the TRPV1 receptor mediates perception in two different modalities. Two stimuli that are sensed by the same molecular receptor will result in the same neuronal activity and therefore in the same phenomenal character; but the phenomenal-character criterion would judge heat and capsaicin to be two stimuli in the same modality. However, what is represented by the stimuli is a botanical compound in one case and temperature in the other. There are other examples of receptors that are sensitive to different types of stimuli for which the same analysis applies (Dhaka et al., 2006).

Since the four criterion-based approaches come to contradictory results outside of vision and audition, they cannot all be correct and philosophers have argued over which approach is the correct one. Instead of contributing to this debate, we will introduce here an alternative to criterion-based modality individuation. We propose to individuate modalities using the same type of behavioral tests used to construct QSs: forced-choice discrimination tasks. Suppose you have two stimuli. What you want is to see whether the two can be manipulated (altered gradually so as to be more similar) so that in the end they are JND, and then make them a bit more similar so that they come to be totally indistinguishable. If so, then the two original stimuli are accessible by the same modality. If they cannot be made JND and then made to match, then they were not the same modality to begin with. This appeals to discriminative responses to stimuli made problematic by, e.g., the TRPV1 receptor; it is not an appeal to the physical nature of the stimuli themselves. Since capsaicin cannot be gradually altered to turn into heat, TRPV1 mediates perception in two different modalities according to the JND-method.

The JND-method allows individuating senses sensitive to light, sound, temperature, pressure, magnetic field, and electric field. What all these stimuli have in common is that they can be gradually altered by arbitrarily small steps, thereby providing a basis for the JND-method. Most stimulus types represent a continuum like wavelength or temperature that is amenable to this treatment. Chemical stimuli may seem to be an exception because chemical stimuli consist of discrete molecules. Molecules cannot be altered by arbitrarily small steps, instead, the smallest gradual change to a molecule is to add or remove one atom or to replace one atom with a similar atom. In almost all cases, a molecule can be distinguished by smell from the chemically most similar molecule (Laska and Teubner, 1999a, b). However, as discussed in detail in the above section on constructing the olfactory QS, the olfactory stimulus space consists predominantly of mixtures of molecules and the ratios of the components of a mixture can be altered in arbitrarily small steps. Two odorous molecules A and B are connected by JND steps through mixtures of A and B with different ratios of the two components. The JND-method of modality individuation can therefore also be applied to the chemical senses much in the same way in which it can be applied to all other senses.

It has to be pointed out, however, that applying the JND-method of modality individuation requires a prior decision on what stimuli to include as components of mixtures. If one allows mixing of, for example vanillin (a tasteless odorant) and sugar (an odorless tastant), then odor and taste will be individuated as a single modality by the JND-method, if, as can be expected, orally administered mixtures of vanillin and sugar can be gradually altered from pure vanillin to pure sugar along a line of indistinguishable mixtures of different ratios. If one prevents mixing of tastants and odorants then odor and taste will be individuated as two modalities. If chemical stimuli are allowed to be mixed with touch and temperature stimuli inside the oral cavity, the JND-method will individuate the multisensory modality called “flavor” (Taylor and Roberts, 2004; Shepherd, 2011; Small and Green, 2012).

In summary, when the JND-method of modality individuation is applied, the same method that is used to constructing QSs can be used to individuate modalities. This approach, which individuates modalities through behavioral tests, provides an empirical alternative to the traditional criterion-based approaches. It can be readily applied to senses sensitive to continuous stimuli like light, sound, temperature, pressure, magnetic field, and electric field. To apply it to the chemical senses, it has to be supplemented by a limit on the stimuli that can be mixed. JNDs fix the mental qualities specifically and exclusively by appeal to the perceptual role that states with those mental qualities play, independent of whether the states are conscious states. So if we are to find a supplement to JNDs that puts limits on the mixing of stimuli for purposes of constructing the relevant QS, we would want to explore possibilities that appeal in one way or another to the perceptual role of states with the relevant mental qualities, independent of whether those states are conscious. Any such supplement to JNDs would conform the spirit of QST.

Because the JND method of modality individuation requires behavioral experiments, the outcome of applying this method is at this point speculative. It therefore remains to be seen how the outcome of this approach compares to the outcomes of the criterion-based approaches. However, regardless of the results, the JND method has two advantages over the traditional approaches. First, the same method that is used to individuate the senses is used to construct QSs of the perceptual qualities in the individual senses. This is an elegant way of arranging all sensory qualities in just a single step. Second, the JND method is based on an empirical procedure and it is therefore transferable to any situation. Unusual senses (echolocation, polarized light, tactile vision, etc.) or unusual subjects (aliens, synesthetes, etc.) that often require special treatment by the criterion-based approaches therefore pose no special problem for the JND-method of modality individuation.

How a modality is individuated has, of course, important consequences for the features attributed to it. For example, how olfaction is individuated determines whether it exhibits a spatial aspect, perhaps calling for an independent QS that specifically determines olfactory spatial mental qualities. It is commonly thought that smells seemingly just appear within our nostrils or as undifferentiated transparent odorous clouds within our surroundings. This has a led a large number of philosophers to argue that olfactory perception does not represent the location or direction of olfactory stimuli (Lycan, 2000; Smith, 2002; Matthen, 2007; Peacocke, 2008; Batty, 2010). Almost all odors activate at suitable concentrations both the first cranial nerve (the olfactory nerve) and the nerve endings of the fifth cranial nerve (trigeminal nerve) in the nasal cavity (Doty and Cometto-Muñiz, 2003). What types of spatial aspects olfaction exhibits depends on whether what is mediated by the trigeminal nerve is considered to belong to the olfactory modality.

Even if one considers olfaction to be only what is mediated by the olfactory nerve, these philosophers may be mistaken. Humans can locate a smell using differences in concentration. Locating the source of a smell requires active exploration; movement of the whole body or at least of the head (Richardson, 2011). In this respect, locating an odor source is similar to locating a heat source (Smith, 2002). Olfactory experience can, across time (diachronically), have spatial structure, although it can be debated if this structure is represented in perception or cognitive. At any particular time (synchronically), olfactory experience has no spatial structure.

If one individuates modalities in a way so that the trigeminal nerve contributes to the olfactory percept (as the JND method does), then olfaction also presents us synchronically with spatial properties, in a similar fashion to audition, in which comparisons between the inputs into the two ears supports locating sound sources. Although it has been shown that for stimuli that activate only the olfactory nerve subjects cannot tell if the odor is in the left or right nostril (Radil and Wysocki, 1998; Frasnelli et al., 2008), this is easily possible for stimuli that activate the olfactory and the trigeminal nerve (Kleemann et al., 2009). This enables us to determine the location of the odor source because there are small differences in timing and intensity of the stimulus between the two nostrils that enable us to locate odorants within 7–10 degree of their location (von Bekesy, 1964). Further evidence supporting the claim that each nostril creates a different olfactory percept is substantiated by Zucco and Chen (2009, p. 1564), who demonstrate that binaural rivalry exists between the nostrils, such that “alternating odor percepts [occur] when two different odorants are presented to the two nostrils.” The difference between the perceptible properties presented to each nostril has also been shown to allow us to track an olfactory stimulus through an environment over time (Porter et al., 2007).

Thus, whatever the QS may be for olfactory qualities, the spatial perceptible properties of olfaction will require an additional QS dedicated specifically to reflect the JNDs of spatial location of olfactory perceptible properties. Olfaction does present spatial perceptible properties that can be ascertained in accordance with JND judgments. The percept mediated by the olfactory nerve does so diachronically and the percept mediate by the trigeminal nerve synchronically. Those who deny any spatial aspect to olfactory experience are simply mistaken. Olfactory perception does present objects with perceptible spatial properties, but they are diffused across the environment in a manner that is dissimilar to the way the spatial aspects of objects are presented to vision. Odors have spatiotemporal perceptible properties, yet locatedness might not be a perceptible olfactory property that makes it unlike vision. Whatever the case about spatial aspects of olfaction, however, there is no conflict with QST, since the theory allows for different types of QS for different modalities in general, and so also in respect of spatial properties.

## EXPERIENCE-DEPENDENT OLFACTION

As noted in the introductory section on QST, the theory predicts that the space of mental qualities is enhanced or made more fine-grained by improvements in perceptual acuity. The acuity of our perceptual discriminations themselves, independent of conscious awareness, can improve either through maturation of the perceptual system itself or by way of perceptual learning. These processes lead to an enlargement or more fine-grained development of the QS, and enable us to make a greater number of perceptual discriminations. And QST posits that the ability to make more perceptual discriminations is due to the occurrence of a correspondingly greater number of mental qualities.

In this section we explore what is currently known about the processes by which olfactory acuity is improved through perceptual learning in application to QST. The evidence surveyed discounts the role of maturation, but supports the claim that the enhancement of perceptual acuity need not depend upon or even be accompanied by, consciousness or subjective awareness. Our subjective awareness of the contents of perception might well, sometimes at least, be modulated and even enhanced by our conceptual repertoire and descriptive resources, since we can often report on more nuanced aspects of our experiences as we acquire more fine-grained conceptual resources. But since QST defines the mental qualities independent of subjective awareness of them, the enhanced ability to report arguably reflects only subjective awareness of the qualities, and not the mental qualities themselves (e.g., Rosenthal, 2005, p. 187). In what follows we show that these claims are perfectly in keeping with what is known about experience-dependent olfaction based on the studies survey below that enhanced olfactory acuity need not depend upon subjective awareness or an increase in conceptual repertoire and descriptive resources.

There is some indication that enhanced perceptual acuity in olfaction supports the claim by QST that an enhancement of the QS independent of any subjective awareness can enhance our discriminative abilities. An increased presentation of an odorant even subliminally can generate further olfactory abilities for detecting and discriminating that stimulus from others. Wysocki et al. (1989) demonstrated that merely increasing the presentation of a stimulus enables a subject to gain the ability to detect and discriminate an odorant they were previously unable to smell at all. Thus, increased exposure to an odorant that one could not subjectively report smelling yielded a larger QS and thereby enhanced perceptual acuity in detecting and discriminating the odorant that is in keeping with the evidence, about to be surveyed below, from the enhanced perceptual acuity of perfume workers and the sensory training of wine experts.

Olfactory acuity is not always enhanced by the number of experiences one undergoes or by increased exposure to an odorant. Indeed, a subject’s ability to discriminate between odorants can be adversely effected by increased exposure to the binary mixture of these odorants, such that familiarity with the mixture decreases the subject’s ability to discriminate the components odorants (Case et al., 2004). These results might be attributed to so-called acquired equivalence, in which two odors that are judged similar and that frequently co-occur become increasingly difficult to discriminate (Stevenson and Boakes, 2003). Acquired equivalence is of relevance to QST, since it suggests a reduction in the number of distinct olfactory mental qualities resulting from decreased ability to discriminate odorant stimuli. This arguably shows that not only does improvement in perceptual acuity lead to an enhanced QS of mental qualities, but a decrease in discriminative perceptual abilities is reflected in a reduction in the fineness of grain of the QS of olfactory mental qualities.

Olfaction that is influenced by experience is especially fascinating with regards to QST claims regarding enhanced perceptual acuity, since linguistic tags and semantic resources are known to play only a limited role in improving olfactory acuity. Olfactory perception and discriminative ability are enhanced primarily through an increased number of stimulus presentations and olfactory experiences, and linguistic tags and linguistic resources for describing olfactory experiences play only a limited role in improving olfactory discriminative ability. This fits well with QST, which defines mental qualities independent of subjective awareness; linguistic tags and descriptive resources would presumably enhance only the subjective awareness of the olfactory mental qualities, and not the mental qualities themselves, which are fixed just by discriminative ability independent of subjective awareness.

However, it should be noted that in what follows none of the studies surveyed below employed forced-choice discrimination tasks using subliminal stimuli. Thus, their results do not directly bear on the claim by QST that mental qualities are determined according to judgments of JND that can occur independent of conscious awareness of stimuli. Rather, the literature below on consciously mediated enhanced olfactory perceptual acuity is surveyed below for the sake of completeness. Furthermore, these studies are instructive as it is arguable that if in adult testing conscious olfactory acuity is only slightly influenced by linguistic tags and semantic resources, then the same should hold for non-conscious olfactory acuity. Additional research needs to be conducted to confirm that conclusion, by examining whether these same results do occur in the discrimination of subliminally presented olfactory stimuli.

The process of maturation is unlikely to be a major influence in the increased fineness of grain of olfactory perceptual acuity, since the olfactory QS is relatively consistent from age three through old age. The olfactory system is fully developed and functional *in utero* and is responsible for an infant’s ability to identify its mother (Russell, 1976; Porter and Winberg, 1999), as well as the ability to distinguish relatives from strangers (Porter et al., 1986). Children’s olfactory capacities are fully developed by age three in terms of odorant detection threshold and hedonic judgments (Stein et al., 1958; Steiner, 1977; Schmidt and Beauchamp, 1988; Schmidt, 1992; Soussignan et al., 1997). There is some difference in detection thresholds, but this is most likely due to adaptation to ecologically important stimuli. Furthermore, while some studies have shown that children’s ability for odor recognition and identification is inferior to that of adults, when linguistic competence and overall vocabulary are controlled for, these apparent differences disappear (Schmidt and Beauchamp, 1988; Lehrner et al., 1999). And though maturation does not enhance acuity, deterioration does play a role in the loss of olfactory perceptual acuity starting at about age 40 (Dulay et al., 2008).

Conscious perception is doubtlessly influenced by our conceptual abilities and linguistic practices that enable us to utilize vocabulary to describe perceptible properties. Conscious olfactory perceptual acuity is influenced by verbal mediation in terms of learned linguistic tags, but also to a large extent by the number of exposures to an odorant. In a classic set of experiments, Rabin (1988) demonstrated that increased exposure to an odorant improved the subject’s ability to discriminate that odor from others. In the first experiment the exposure condition showed an increase discriminative ability as compared to the control, yet the subjects in a further condition in which they learned relevant linguistic tags showed an even greater increase in discriminative ability. The results from the second experiment partially address this by demonstrating that the familiarity of an odorant allowed an enhanced discriminative capacity for similar odorants. But all this could reflect the relevance of linguistic tags to subjective awareness of olfactory mental qualities, and not to the mental qualities themselves, which are on QST fixed independent of any such subjective awareness.

Since perceptual acuity increases even for identity and naming in accordance with familiarity of the odor (Homewood and Stevenson, 2001) and practice (Cain, 1979), it is worth considering how olfactory acuity is mediated by memory. Our almost pathological inability to name odors (Olofsson et al., 2012) has led many to question the format of odor memory. The underlying mechanisms and processes are still being investigated, but a growing body of evidence suggests that olfactory memory is not mediated by linguistic tags or verbal coding. Odor memory is possible without verbal mediation (Møller et al., 2004). Olfactory coding and experiences are non-linguistically formatted and do not dependent on language processing (Goodglass et al., 1968; Herz, 2000). There has been no direct research concerning the format of non-conscious olfactory mental qualities. However, if olfactory memory, which is arguably independent of subjective awareness, does not depend on linguistic tags or verbal coding, then the QS of olfactory mental qualities, which is also independent of subjective awareness, is unlikely to depend on linguistic tags and verbal reports.

Perfume experts do have enhanced olfactory discriminative abilities. That enhanced ability might in part be due to increased descriptive resources or linguistic labels, though the number of experiences has a much greater influence (Gilbert et al., 1998). Perfume experts and novices mostly overlap in their odor categorization, as determined by their sorting of perfumes into groups based on consciously perceived similarities and differences. The perfume experts were more parsimonious in the number of groupings, but the difference was not statistically significant. Moreover, despite the expert’s more exacting usage of linguistic descriptors for odor groups, their groupings themselves were mostly similar to those of the novice consumers. Not only did the perfumers’ enhanced semantic repertoire and linguistic tags show no marked affect in their categorical groupings of odors, but where they did differ in similarity judgments, the differences are best explained by the larger number of times the experts had been previously exposed to the stimulus (Veramendi et al., 2012). Thus, even the slight increase of the experts’ parsimony in the number of groupings, itself not statistically significant, is best attributed to the number of experiences, rather than linguistic sophistication.

Further support for the idea that stimulus exposure leads to and is primarily responsible for an increase in olfactory acuity comes from studies that show that our conscious olfactory discrimination abilities increase with training and exposure. More familiar odors are easier to discriminate than those that are unfamiliar to us (Jehl et al., 1995). Perfume shop workers have an increased ability to discriminate odors, yet their stimulus detection threshold and ability to identify odors from a list of descriptors is not enhanced (Hummel et al., 2004). These results suggest that peripheral sensory plasticity or increased descriptive resources are not the determining factor in this increased discriminative ability. Instead, this increase appears to be driven by some sort of perceptual sensory template. Olfactory memory enables our capacity for perceptual discrimination in a manner that is not linguistically driven, yet in perfect keeping with the claim of QST that perceptual acuity can be improved through a sheer increase in the number of conscious or unconscious experiences that the subject undergoes, thereby enhancing our perceptual ability for making judgments of JND, which results in an enlarged or more fine-grained QS.

That odor acuity improves with an increase in olfactory experiences and training independent of linguistic mediation has also been documented in studies of wine experts. Wine experts outperform novices at odor discrimination (Solomon, 1990; Melcher and Schooler, 1996; Bende and Nordin, 1997) and their increased ability results from greater perceptual skill and not verbal or descriptive resources (Parr et al., 2002). Parr et al. showed that the experts had an enhanced ability to recognize odors, but that they did not outperform novices either in terms of their sensitivity threshold for odorant detection or the verbal memory task. When this result is combined with their findings that odor naming and odor recognition were not positively correlated, it provides further reason to think that increased olfactory recognition and discriminative acuity do not depend on an increased availability of linguistic tags or semantic descriptors.

This does not preclude the possibility that novices are sometimes aided in coming to discriminate odors consciously in more fine-grained ways by learning new tags or descriptive resources for distinct mental qualities (Rosenthal, 2005, chapters 1 and 7). The tags or descriptive resources would be relevant to novices’ subjective awareness of mental qualities, which might well already differ. Further testing based on subliminal presentation of olfactory stimulus using forced-choice methodology or the equivalent is needed.

More recently it was shown that wine experts can more accurately discriminated between two varieties of wines as indicated by their correctly sorting samples into their respective groups (Ballester et al., 2008). Moreover, these results showed an intersubjective convergence of the experts on their judged typicality of each variety of wine, which the authors interpret as indicating that the experts discriminated each kind of wine in respect of its perceptual characteristics. However, these results do not address the question whether the increased perceptual ability and judgments of typicality are caused by an enhanced perceptual strategy that is more analytic and focuses upon the perceptible qualities of the stimulus, as against being due instead to enhanced descriptive repertoire that allows greater conscious discriminative ability. At least one study suggests that it is the former. In this study, wine experts were trained to detect and discriminate between key sensory characteristics using sensory training (Tempere et al., 2012). By exposing experts to key odorous wine compounds Tempere et al. increased the experts’ perceptual abilities by lowering their detection threshold through increased exposure to the key compounds in a fashion that only allowed them further discrimination within that group of qualitatively similar perceptible properties.

Taken together, the results from these studies provide evidence that olfactory acuity improves with the number of olfactory experiences in a manner that does not depend upon maturation of the olfactory system or the nature or richness of linguistic representation of the olfaction qualities. Human discriminative abilities increase in accordance with the overall perceptual QS of olfaction that is evidently not mediated by linguistic or verbal coding. In the wine-training research the overall effect was specific to the training stimulus, which indicates that an enhancement of the olfactory mental-QS is determined by the perceptible properties we can discriminate among.

A key test for the claim that olfactory discriminative acuity is mediated by experience and not linguistic coding or descriptive resources is cross-cultural comparisons of odor perception and categorization. Linguistic conventions and conceptual naming strategies differ between cultures, yet there is great overlap in overall odorant categorization as determined by odorant sorting experiments using consciously judged perceptual similarities (Chrea et al., 2005a,b). In these studies, American, French, and Vietnamese students were shown to sort odor samples into similar groupings that were not consistent with their groupings of the odor labels that would be associated with the olfactory samples presented in the odorant sorting task. Since the odorants were categorized differently from the labels, there is reason to believe that the verbal labels did not determine odorant grouping. Additionally, the differences between the different cultural groups in odor grouping displayed a familiarity effect. Individuals from cultures that were more familiar with an odor categorized it similarly, thereby showing that the number of exposures was the best indicator of olfactory discriminative ability for odor categorization in this type of odorant-sorting task.

Experience-dependent olfactory perceptual abilities pose no problem for QST. However, there is another result that might at first sight seem to do so. Research on olfactory sensitivity, using classical conditioning, has demonstrated that enantiomers can be discriminated by subjects despite their subjective reports that they possess identical olfactory qualities (Li et al., 2008). Li et al. demonstrated that supraliminally indistinguishable optical isomers are discriminable after classical conditioning. According to QST, discriminative ability determines mental quality. In this case there is an increase in discriminative acuity and thus an enhancement of the olfactory QS in respect of discriminative ability, yet no subjective report of any increase in perceived olfactory quality.

But despite initial appearances, this is readily explained by QST, since the mental qualities that discriminative ability determines on the theory are independent of any subjective awareness of those mental qualities. In this case we have increased refinement of mental qualities that is not reflected in a corresponding ability to distinguish those qualities. Thus in the next section we turn to non-conscious olfactory qualities and to whether we have good reason to think there are non-conscious mental qualities corresponding to each of the enantiomers post-conditioning that we cannot report on.

## OLFACTORY QUALITIES IN NON-CONSCIOUS OLFACTORY STATES

Non-conscious olfactory states that are genuinely qualitative might be inferred from the phenomena of blind smell (Schwartz et al., 1994; Sobel et al., 1999; Schwartz, 2000), mate selection (reviewed in Wilson and Stevenson, 2006), social acquaintance selection (Li et al., 2007), and an argument from absence which would explain the deterioration in quality of life following the onset of anosmia. Anosmia is the most common disorder of olfactory pathology in which individuals lose their sense of smell. In some cases anosmia is due to the presence of a psychological disorder, but the vast majority of cases result from damage to the olfactory bulb due to either infection or head trauma. In addition to their inability to perceive olfactory stimuli, anosmic individuals also experience a decrease in their hedonic quality of life (Miwa et al., 2001; Keller and Malaspina, 2013), which in turn is often causally implicated in the further development of depression (Deems et al., 1991). We are not aware of our olfactory experiences most of the time, but they imbue our lives with a qualitative character of experience, which becomes most striking when it is absent (for a review of all these phenomena and their relation to other theories of non-conscious qualitative states, such as Block, 1993, 1995, 2001, 2007, 2008, 2009) distinction between access and phenomenal consciousness, see “smelling phenomenal” by Young (in press) in this special research topic.

But a concern regarding each of these phenomena as evidence for unconscious olfactory qualitative states is that humans are not commonly aware of their olfactory experience and generally discount their overall olfactory abilities (Sela and Sobel, 2010). So one might question whether we should conclude, in accordance with QST, that non-conscious olfactory perceptual states do have genuine mental qualities.

But it turns out that olfaction can provide independent evidence using secondary processing measures that non-conscious olfactory discriminative ability matches that of conscious discriminative ability, thereby corroborating the view of QST that olfactory mental qualities do indeed occur independent of consciousness. They occur not merely in the absence of attention, but in the absence of subjective awareness itself.

Secondary processing measures are traditionally employed in disputes regarding computational implementations of cognitive abilities; but similar measures are also available in the measuring of perceptual states. In addition to a state’s role in enabling discrimination of olfactory stimuli and in addition also to any of distinctive role it may have, there might be other secondary properties we can use to judge whether or not the same type of state occurred, utilizing a very similar if not always wholly identical physical realization. Secondary processes are correlated properties or incidental effects (Cummins et al., 2001), such as speed, error rate, types of errors, or fatigue, etc., of a perceptual system in the performance of particular tasks.

In olfactory research the perceptible property of valence (the perceived pleasant or unpleasant property of an odor) provides just such this type of measure for assessing the veridical nature of this perceived olfactory property, independent of subjective reports based on conscious awareness. Behavioral measures such as sniff rate and volume, response time, and heart rate can all be used as independent measures that indicate the olfactory system is treating pleasant or unpleasant olfactory stimuli in the same fashion, regardless of whether we consciously perceive the odors or can subjectively report upon their olfactory qualitative character or their valence. The perceptible property of olfactory pleasantness or unpleasantness can be employed using sniff rates as a secondary measure to verify the perceived pleasantness or unpleasantness of an odor even when the subjects are subjectively unaware of any relevant olfactory mental qualities.

Humans modulate their sniff rate and volume 150 ms after the onset of a stimulus depending on the odor’s concentration and valence (Johnson et al., 2003). The stimulus-dependent response of human sniffing is such that intense and unpleasant odorant are sniffed less vigorously and with a decreased volume. Measurement of olfactory motor responses to odorants is reliable enough to be used as a non-verbal measure of human’s detection and categorization of the odor (Frank et al., 2003). Additionally, it has been shown that while individuals with fully functional olfactory systems modulate their sniffing in accordance with the valence of the odor, anosmics show no such response (Harland and Frank, 1997). This shows that the sniff response only occurs when the subject perceives the valence of the presented stimulus and can arguably be used to show that the variable of sniff rates can be used to demonstrate the occurrence of mental qualities independent of conscious subjective reports, and hence in the absence of subjective awareness.

Sniff rate and volume are not the only secondary measures for assessing odor valence. Response time is faster in detection and discrimination tasks for unpleasant odors (Bensafi et al., 2003), and heart rate measurements show that we involuntarily react in distinctive ways to unpleasant odors (Bensafi et al., 2002). The subject’s non-conscious perception of odor valence can be verified using behavioral non-verbal measure such as sniff patterns, response time, and physiological response of heart rate. These measures, and in particular the invariance of sniff rate between conscious and non-conscious presentations of an odor, further support QSTs methodology of identifying mental qualities in light of a state’s perceptual role in enabling perceptual discriminations, independent of whether the subject can report undergoing an experience with qualitative character and so independent of subjective awareness.

The sameness of perceptual role of olfactory states in conscious and non-conscious olfaction as assessed by the secondary measures of sniff-rates (as well as other behavioral measures) is what supports the methodology of inferring sameness of perceptual content and mental quality. Thus, these secondary measures can be employed as empirically sound tools for verifying the perceptible property of valence independently of subjective reports.

Secondary processing measures corroborate QSTs conclusion that occurrence of mental qualities can be established independently of subjective awareness. However, further research is required on the sniffing parameters of subjects during judgments of JNDs, since olfactory imagery generates consciously imagined percepts. In addition, of the aforementioned phenomena that provide evidence for non-conscious olfactory states that are genuinely qualitative, only the work of Schwartz et al. (1994) on blind smell employed a forced-choice discrimination task, but without measurements of sniffing patterns. Nonetheless these further measures provide a promising way to strengthen the extrapolation of the QS of perceptible JNDs to the QS of mental qualities, since they provide further motivation that the best explanation must involve the existence of non-conscious mental qualities. The alternative explanation that non-conscious olfactory states do not exhibit qualitative character is unmotivated, because behavioral and physiologically measures can indicate that at least for the perceptible property of valence the olfactory system is treating certain non-conscious olfactory states as equivalent to those that exhibit conscious qualitative character.

Even employing secondary measures, the aforementioned Li et al. (2008) study provides what appears superficially to be a problem for QST. Their findings indicate that even though the subjects can be trained to discriminate between enantiomers with identical olfactory qualities, the subjects’ ratings of the valence, intensity, and familiarity of these structures were not affected. Training might have produced the ability to detect the differences in the enantiomer’s structure, such that subjects could discriminate between the enantiomers, but it did not lead to a reportable change in the qualitative character of the olfactory quality of the conditioned enantiomer. Furthermore, secondary processing measures indicate that even after training the perceptible properties of each enantiomer remained the same. These results may seem to run contrary to what QST would predict, since the increased discriminability should yield further mental qualities.

However, QST defines mental qualities independent of conscious awareness of olfactory stimuli, in terms simply of discriminative ability, whether conscious or not. So Li et al.’s results do not after all threaten QST. It is perfectly possible that mental qualities occur non-consciously in a more fine-grained way than we are subjectively aware of, and hence in a more fine-grained way than we can report.

Indeed, as Rosenthal (2005, ch. 7, 2010) has argued, that is very likely the best explanation for the way novices can quickly learn to distinguish consciously previously indistinguishable stimuli, such as two wines or two musical instruments, when given terms to attach to the two stimuli. Having distinct terms for the two experiences, the experiences come to be consciously distinct. The best explanation is that distinct mental qualities already occur on tasting the two wines or hearing the two instruments, but only non-consciously. Learning the new terms facilitates those unconscious mental qualities’ becoming conscious. Li et al.’s fascinating research on enantiomers would present a challenge to QST only if the theory implied that discriminative ability in the non-conscious case is always matched in the conscious case. But the theory does not imply that, and indeed implies that the opposite is likely.

## CONCLUSION

QST fixes and describes the mental qualities for each perceptual modality by appeal to the discriminative ability that yields JNDs, independent of whether the relevant perceptual states are conscious. Such JNDs among perceptible properties allow the construction of a QS of those properties. Because the perceptual states that discriminate perceptible properties differ in mental quality, a QS of discriminable perceptible properties serves also to fix the mental qualities that are operative in such discrimination.

The construction of a QS for the visual mental qualities is relatively straightforward. The construction of a QS that would do justice to olfactory stimuli, however, poses special challenges. This is due in part to the vastly greater number of olfactory discriminations we can make compared to those we can make, for example, among visible stimuli. The challenge for constructing an olfactory QS becomes especially formidable in connection with mixtures of olfactory stimuli. Because of these and related factors, the olfactory QS will inevitably have a huge number of dimensions. Nonetheless, there is no reason to doubt that a QS can be constructed that would capture the ability to discriminate among olfactory stimuli, and thereby the olfactory mental qualities that underlie that ability.

A question arises also about what distinguishes olfactory stimuli from stimuli of other types and, more generally, about how to distinguish each perceptual modality from others. It turns out that QST helps here as well; the same appeal to discriminative ability that fixes the mental qualities can be harnessed to distinguish the various modalities. This way of distinguishing the modalities, moreover, is arguably superior to more traditional ways of doing so. And the QS methodology has the additional benefit of allowing a QS treatment of mental qualities that figure specifically in the sensing of location, as well as perceptible size and shape for those modalities that enable the discrimination of those spatial perceptible properties. Moreover, it provides the tools to confirm just which spatial properties each modality can discriminate.

QST accommodates the perceptual learning and maturation known to occur with olfaction and other modalities. Such processes result in the enhancement of the relevant QS, either by expanding it or by increasing its fineness of grain. This applies both to conscious changes in olfactory discriminative ability and to non-conscious development. But it is likely that an increased linguistic repertoire for olfactory experiences and the acquisition of new linguistic tags to refer to them enhances the way we are consciously aware of olfactory stimuli, but not the underlying ability to discriminate among olfactory stimuli. QST predicts this, since it fixes mental qualities independent of their being conscious and also independent of such linguistic tags and repertoire.

QST proceeds independently of whether the mental qualities are conscious because the theory fixes and describes mental qualities by their role in discriminating among barely discriminable stimuli, and such discriminations occur both consciously and not. Mental qualities are posited to explain the differences among perceptual states that enable such discriminations, whether or not those discriminations are conscious. In the conscious case, there is in addition first-person access to those mental qualities, but QST fixes the mental qualities solely by their role in discrimination, independent of such first-person access. So the theory readily accommodates mental qualities that are not conscious. And olfaction provides compelling independent corroboration of non-conscious olfactory mental qualities by appeal to the secondary processing measures of non-conscious olfactory perception.

In conclusion, QST provides a powerful theoretical tool for the understanding and the scientific study of olfactory mental qualities and olfactory perception. Olfaction in turn provides further evidence in support of QST, which has now been shown to be consistent with empirical findings in different modalities.

## AUTHOR CONTRIBUTIONS

All authors wrote the paper together.

## Conflict of Interest Statement

The authors declare that the research was conducted in the absence of any commercial or financial relationships that could be construed as a potential conflict of interest.
